# Molecular Characterization Related to Ovary Early Development Mechanisms after Eyestalk Ablation in *Exopalaemon carinicauda*

**DOI:** 10.3390/biology12040596

**Published:** 2023-04-14

**Authors:** Shaoting Jia, Jitao Li, Jianjian Lv, Xianyun Ren, Jiajia Wang, Qiong Wang, Ping Liu, Jian Li

**Affiliations:** 1 National Key Laboratory of Mariculture Biobreeding and Sustainable Goods, Yellow Sea Fisheries Research Institute, Chinese Academy of Fishery Sciences, Qingdao 266071, China; 2 Function Laboratory for Marine Fisheries Science and Food Production Processes, Qingdao National Laboratory for Marine Science and Technology, Qingdao 266071, China

**Keywords:** *Exopalaemon carinicauda*, eyestalk, ovary, hepatopancreas, vitellogenesis

## Abstract

**Simple Summary:**

*Exopalaemon carinicauda* is a typical representative of crustaceans. However, the mechanism of ovary early development remains obscure. The purpose of this study is to identify and verify important genes related to ovary development by transcriptome sequencing and two-color fluorescent in situ hybridization, respectively. The result would provide clues for further study of ovary development in crustaceans.

**Abstract:**

Eyestalk ablation is an effective method to promote ovarian development in crustaceans. Herein, we performed transcriptome sequencing of ovary and hepatopancreas tissues after eyestalk ablation in *Exopalaemon carinicauda* to identify genes related to ovarian development. Our analyses led to the identification of 97,383 unigenes and 190,757 transcripts, with an average N50 length of 1757 bp. In the ovary, four pathways related to oogenesis and three related to oocyte rapid growth were enriched. In the hepatopancreas, two vitellogenesis-associated transcripts were identified. Furthermore, short time-series expression miner (STEM) and gene ontology (GO) enrichment analyses revealed five terms related to gamete generation. In addition, two-color fluorescent in situ hybridization results suggested that *dmrt1* might play a vital role in oogenesis during the early stage of ovarian development. Overall, our insights should support future studies focusing on investigating oogenesis and ovarian development in *E. carinicauda.*

## 1. Introduction

In crustaceans, the ovary is a key reproductive organ considering that effective spawning is the primary factor for embryogenesis. Therefore, elucidating molecular mechanisms underlying ovarian development in crustaceans is vital for the growth and development of the aquaculture industry.

Ovarian development in crustaceans involves six main processes: oogonium, meiotic prophase, previtellogenesis, primary vitellogenesis, secondary vitellogenesis, and oocyte maturation [1]. In lower crustaceans, such as branchiopod crustaceans, ovarian development is similar to that in lower insects owing to the presence of nurse cells [2]. However, as higher crustaceans do not contain nurse cells, oogonia differentiate into a large number of oocytes, which increases the frequency of spawning [3]. Oocyte growth is slow during the primary stage of oogonium proliferation and differentiation, with rapid growth mainly occurring during vitellogenin (VTG) synthesis [4]. In some crustaceans, the ovary and the hepatopancreas are chiefly involved in vitellogenesis [5]; however, the initial signal underlying this process remains unclear.

The crustacean’s neuroendocrine system plays an important role in regulating ovarian development. Pertinent studies have focused on the X-organ–sinus gland complex (XO-SG), which represents a complex neuroendocrine regulatory network [6]. This complex is located in the eyestalk and secretes five major hormones—red pigment-concentrating hormone (RPCH), pigment-dispersing hormone (PDH), crustacean hyperglycemic hormone (CHH), molt-inhibiting hormone (MIH) and gonad-inhibiting hormones (GIH). In female crustaceans, GIH prevents vitellogenin synthesis by inhibiting the insertion of leucine in the hormone formation. Accordingly, GIH is also known as vitellogenesis-inhibiting hormone [7]. MIH, a key regulator of molting and reproduction in crustaceans, is involved in inhibiting molting as well as inducing ovarian maturation [8,9]. Methyl farnesoate, which is synthesized by the mandibular organ of crustaceans, stimulates oocyte growth, increases VTG expression levels in the hepatopancreas, and promotes ovarian development. Mandibular organ-inhibiting hormone evidently inhibits the function of methyl farnesoate [10]. Further, gonadal-stimulating factors secreted by the thoracic ganglion promote ovarian development [11]. Based on the mechanism via which the crustacean neuroendocrine system is regulated, eyestalk removal has been reported to accelerate ovarian development and increase fecundity [12]. However, functional genes and molecular mechanisms related to ovarian development remain largely unknown, mainly because efficient cell manipulation and gene editing techniques are still lacking for crustaceans.

*Exopalaemon carinicauda* is an economically important shrimp species in China. It is popular among consumers as its meat not only is delicious but also shows high protein and low fat content. As an experimental animal, *E. carinicauda* shows several advantages, including a small transparent body and short reproductive cycle. *E. carinicauda* was the first reported species of decapod crustaceans that was suitable for gene editing [13]. It thus became a model organism in crustacean research focusing on ovarian development. Nevertheless, only few studies have explored ovarian development and regulatory mechanisms in *E. carinicauda*.

Herein, we applied transcriptome sequencing to analyze the gene expression profile in *E. carinicauda* ovary and hepatopancreas tissues at 1, 6, and 11 days after eyestalk ablation. Data analyses led to the identification of some differentially expressed genes (DEGs) and significantly enriched pathways as well as gene ontology (GO) terms. Furthermore, the expression trend of DEGs in ovary and hepatopancreas samples was evaluated through short time-series expression miner (STEM) analysis. Finally, real-time PCR and two-color fluorescent in situ hybridization were performed to validate the expression of key DEGs.

## 2. Material and Methods

### 2.1. Ethics

This animal study was reviewed and approved by the Ethics Committee of the Yellow Sea Fisheries Research Institute. Written informed consent was obtained from animal owners.

All experiments were performed in accordance with the Guidelines for the Care and Use of Laboratory Animals in China. This study was approved by the Institutional Animal Care and Use Committee of the Yellow Sea Fisheries Research Institute, Chinese Academy of Fishery Sciences (Qingdao, China; approval No.: IACUC-20190314).

### 2.2. Animals and Sample Collection

*E. carinicauda* was obtained from RiZhao Haichen Aquaculture Co., Ltd. Rizhao City, China. The animals were fed in our laboratory for 2 weeks and cultured in the presence of abundant oxygen at 25 °C and 30‰ salinity. Live healthy shrimps with undeveloped ovaries were divided into two groups: experimental and control. Each group included 36 female shrimps. In the experimental group, each shrimp was disinfected after left eyestalk ablation. Ovary and hepatopancreas tissues were collected 1, 6, and 11 days after eyestalk ablation. At each timepoint, three parallel samples were taken for the experimental and control groups, respectively.

### 2.3. RNA Extraction, Purification, and Quantification

Total RNA was extracted from ovary and hepatopancreas tissues with TRIzol (Invitrogen, 15596026, Carlsbad, CA, USA), according to manufacturer instructions. Genomic DNA was removed by DNase I treatment. RNA purity and concentration were determined by Bioanalyzer 2100 (Agilent Technologies, Inc., Santa Clara, CA, USA) and ND-2000 (NanoDrop Thermo Scientific, Wilmington, DE, USA), respectively.

### 2.4. Library Preparation and Sequencing

High-quality RNA samples (OD_260/280_ = 1.8–2.2, OD_260/230_ ≥ 2.0, RIN ≥ 8.0, 28S:18S ≥ 1.0, total of >1 μg) were used to construct sequencing libraries with the Illumina TruSeq^TM^ RNA Sample Preparation Kit (San Diego, CA, USA). All steps, including RNA purification, reverse transcription, library building, and sequencing, were performed by Shanghai Majorbio Bio-pharm Biotechnology Co., Ltd. (Shanghai, China). poly(A) mRNA was purified by oligo-dT-attached magnetic beads, and 300-bp fragments were obtained using a fragmentation buffer. cDNA was synthesized using a SuperScript^®^ Double-Stranded cDNA Synthesis Kit (Invitrogen, 11917020, Carlsbad, CA, USA) with random hexamer primers (Illumina), as per manufacturer instructions. The cDNA target fragment (200–300 bp) was amplified by Phusion DNA polymerase (New England Biolabs, Boston, MA, USA) for 15 PCR cycles. After quantification by TBS380, all RNAseq libraries were sequenced on the Illumina HiSeq X Ten/NovaSeq 6000 sequencer (Illumina, San Diego, CA, USA) to generate 2 × 150 bp paired-end reads.

### 2.5. De Novo Assembly and Annotation

Raw paired-end reads were trimmed by SeqPrep (https://github.com/jstjohn/SeqPrep accessed on 9 September 2021) and quality controlled by Sickle (https://github.com/najoshi/sickle accessed on 9 September 2021) using default parameters. Clean data were then assembled de novo with Trinity (http://trinityrnaseq.sourceforge.net/ accessed on 9 September 2021). All assembled transcripts were aligned with six databases according to sequence similarity: NCBI protein non-redundant (NR), COG, Swiss-Prot, Pfam, GO, and the Kyoto Encyclopedia of Genes and Genomes (KEGG). BLAST2GO (http://www.blast2go.com/b2ghome accessed on 9 September 2021) was used to annotate transcripts based on biological processes, molecular functions, and cellular components. Pathway analysis was performed using KEGG (http://www.genome.jp/kegg/ accessed on 9 September 2021).

### 2.6. Differential Expression and Functional Enrichment Analyses

To identify DEGs, the expression level of each transcript was calculated using the transcripts per million reads method. Gene abundance was determined by RSEM (http://deweylab.biostat.wisc.edu/rsem/ accessed on 15 September 2021). Differential expression analysis was performed using DESeq2. Significant DEGs were those that met these criteria: Q value ≤ 0.05, DEGs with |log_2_FC| > 1 and Q value ≤ 0.05 DESeq2/Q value ≤ 0.001 (DEGSeq). Goatools (https://github.com/tanghaibao/Goatools accessed on 15 September 2021) was used to identify which DEGs were significantly enriched in GO terms, including biological processes, molecular functions, and cellular components. KOBAS (http://kobas.cbi.pku.edu.cn/home.do accessed on 15 September 2021) was applied for KEGG pathway enrichment analysis and metabolic pathways at Bonferroni-corrected *p* ≤ 0.05 compared with the whole-transcriptome background.

### 2.7. Time-Series Analysis

Time-series analyses were performed to detect the profiles and change patterns of DEGs. The STEM (http://www.cs.cmu.edu/~jernst/stem/ accessed on 25 September 2021) clustering algorithm was applied for temporal expression trend analysis DEGs in of ovary samples. The software mimicked the 10 most possible trends, calculated the correlation coefficient for each gene with these preset trends, and ultimately grouped each gene into its most similar trend. Similarly, the time sequence difference analysis of DEGs in hepatopancreas samples was performed using maSigPro (http://www.bioconductor.org/packages/release/bioc/html/maSigPro.html accessed on 25 September 2021). The main steps included determining the regression model, identifying significant genes, finding significant differences, and obtaining significant gene sets.

### 2.8. Real-Time PCR

To validate the accuracy of transcriptome data, the expression of DEGs was verified by real-time PCR. Total RNA was extracted, as per manufacturer instructions. After genomic DNA removal by DNase I, cDNA was synthesized using the ReverTra Ace reverse transcriptase kit (Toyobo, FSK-100, Osaka, Janpan) and random primers. Gene expression was quantified by real-time PCR using the SYBR Green Mix (Toyobo, QPK-201), according to manufacturer instructions; the reaction mixture comprised 10 µL 2 × SYBR Green Real-Time Master Mix, 2 µL cDNA, 0.4 µL forward and reverse primers each, and 7.2 µL H_2_O. The cycling conditions were as follows: 95 °C for 3 min; 45 cycles × (95 °C for 15 s; 55 °C for 20 s; 72 °C for 30 s); 65 °C for 0.06 s; and 95 °C for 0.5 s. Primers were designed by Primer5 and sequences are listed in Table S4.

### 2.9. Two-Color Fluorescent In Situ Hybridization

Two-color fluorescent in situ hybridization was performed to analyze *dmrt1* (encoding double sex and mab-3-related transcription factor 1) expression in ovary samples using a previously reported method [14]. Samples of the ovary at different stages were fixed for overnight in 4% paraformaldehyde and then dehydrated in 30% sucrose. After being embedded in O.C.T. compound (SAKURA, 4583), they were sectioned by a freezing microtome (Leica, CM1950, Wetzlar, Germany). Probes were designed and synthesized according to the cDNA sequence of *vasa*, *dmrt1* and *cyp307a1*, and labeled with digoxin (Roche Diagnostics, 11277073910, Penzberg, Germany) and fluorescein (Roche Diagnostics, 11685619910, Penzberg, Germany), respectively. They were then purified using a kit (Sigma, S5059-70EA, Darmstadt, Germany). The sections were subjected to prehybridization, permeabilization, hybridization, incubation with antibodies, staining, and finally reaction termination. The nucleus was stained with DAPI. Fluorescence images were photographed with a laser confocal microscope (Leica, Sp8, Wetzlar, Germany). Primer sequences used for synthetic probes are listed in Table S4.

## 3. Results

### 3.1. De Novo Assembly of E. carinicauda Ovary Transcriptome

**Figure 1:**
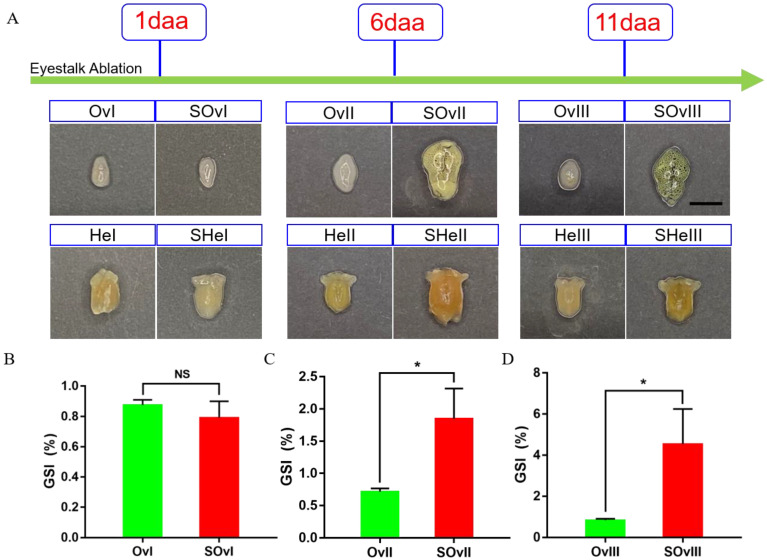
Development of the ovary and the hepatopancreas after unilateral eyestalk ablation in *E. carinicauda*. (**A**) Ovaries and hepatopancreases at different timepoints (scale, 500 μm). (**B**) Gonadosomatic index (GSI) of *E. carinicauda* at 1 day, (**C**) 6 days, and (**D**) 11 days after eyestalk ablation (NS: no significance, * *p* < 0.05). The experimental groups of ovaries include *SOvI*, *SOvII* and *SOvIII*, which means the ovary sample at 1 day, 6 days and 11 days after eyestalk ablation, respectively. The corresponding control groups at the same timepoint were marked as *OvI*, *OvII* and *OvIII*. The experimental groups of the hepatopancreas include *SHeI*, *SHeII* and *SHeIII*, which means the hepatopancreas sample at 1 day, 6 days and 11 days after eyestalk ablation, respectively. The corresponding control groups at the same timepoint were marked as *HeI*, *HeII* and *HeIII*. daa: days after eyestalk ablation.

We obtained 250.96 Gb raw data from 33 samples exposed to different treatments at different timepoints (Figure 1). Additionally, we could observe that the ovary was becoming more mature from 1 day to 11 days after eyestalk ablation. The gonadosomatic index was also becoming bigger which meant the ratio of the ovary weight to the whole body increased (Figure 1B). Raw data were uploaded to the NCBI SRA database (accession No.: SUB11822808). After RNA sequencing and data filtering, 244.65 Gb clean data were obtained. On average, approximately 6.26 Gb data were obtained from each sample, with Q30 percentage > 92.12% (Table S3). Clean data were assembled with Trinity, and the optimized evaluation of assembly results revealed 97,383 unigenes and 190,757 transcripts, with an average N50 length of 1757 bp. The clean reads were aligned to reference sequences obtained from Trinity assembly, and the alignment rate for this analysis was 63.33–78.23%. Additionally, the annotation of the transcripts by six databases were shown in Supplementary Material Tables S2 and S5.

### 3.2. Identification of DEGs

**Figure 2:**
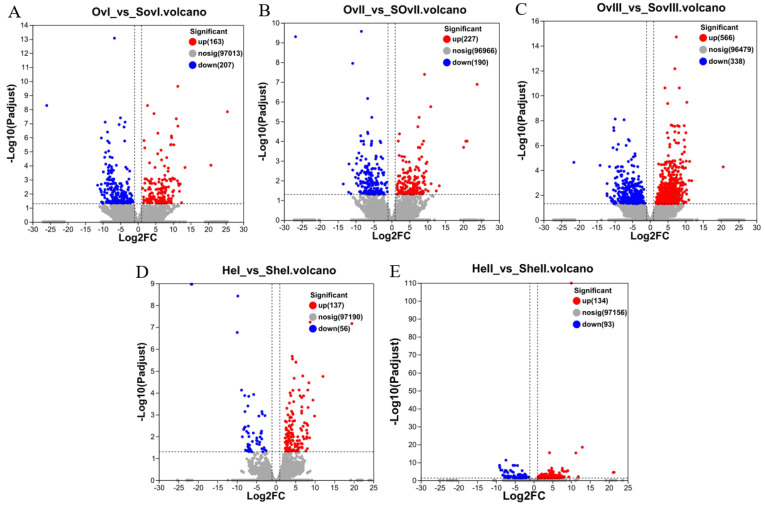
Volcano plots of DEGs. (**A**) Volcano plot of DEGs identified from *OvI_vs_SOvI*, (**B**) *OvII_vs_SOvII*, (**C**) *OvIII_vs_SOvIII*, (**D**) *HeI_vs_SHeI*, and (**E**) *HeII_vs_SHeII*. Red and blue dots indicate significantly up- and downregulated transcripts, respectively. Gray dots represent transcripts with non-significant differences.

DEGs were screened using the following criteria: FDR ≤ 0.05 and |log_2_FC| > 1. In total, 1611 and 710 DEGs were identified from ovary and hepatopancreas tissues, respectively (Figure 2). There were three groups related to the ovary: *OvI_vs_SOvI* included 370 DEGs (163 up- and 207 downregulated), *OvII_vs_SOvII* included 417 DEGs (227 up- and 190 downregulated), and *OvIII_vs_SOvIII* included 904 DEGs (566 up- and 338 downregulated). In addition, there were two groups related to the hepatopancreas: *HeI_vs_SHeI* included 193 DEGs (137 up- and 56 downregulated) and *HeII_vs_SHeII* included 227 DEGs (134 up- and 93 downregulated). Due to the lack of samples in *HeIII* groups, the DEG result of *HeIII* vs. *SHeIII* was not shown.

### 3.3. GO Terms and KEGG Pathway Analysis of Ovary Samples

**Figure 3:**
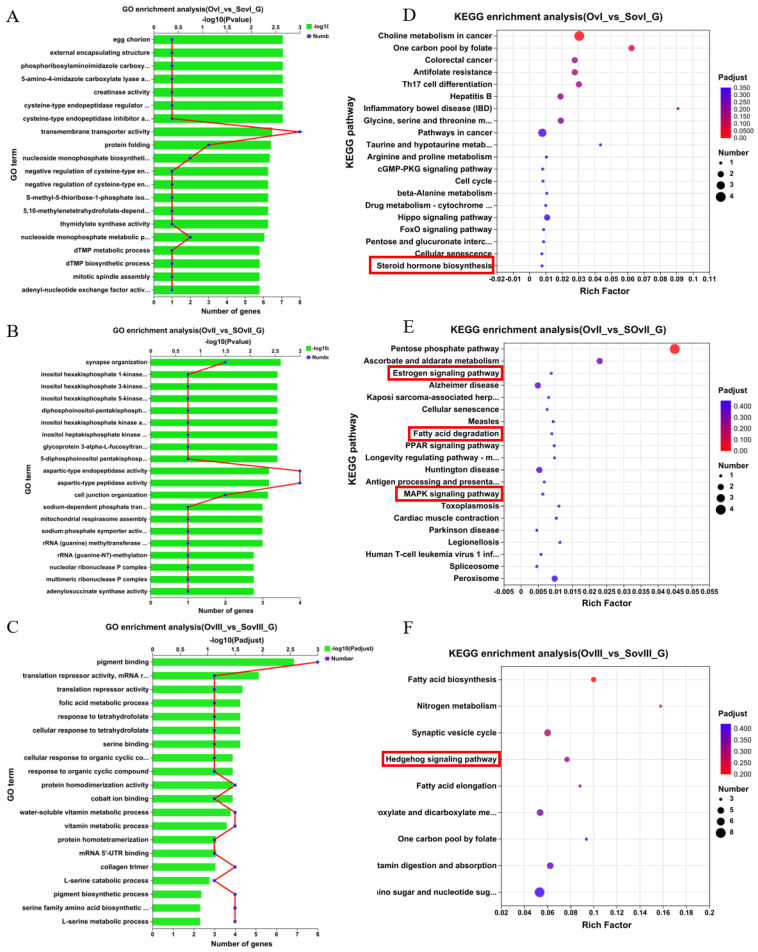
GO and KEGG pathway enrichment analyses of non-redundant transcripts in the ovary after unilateral eyestalk ablation in *E. carinicauda*. (**A**) Top 20 enriched biological processes in *OvI_vs_SOvI*, (**B**) *OvII_vs_SOvII*, and (**C**) *OvIII_vs_SOvIII*. (**D**) Top 20 significant enriched KEGG pathways in *OvI_vs_SOvI*. (**E**) Top 20 significant enriched KEGG pathways in *OvII_vs_SOvII*. (**F**) Top 20 significant enriched KEGG pathways in *OvIII_vs_SOvIII*.

Figure 3A showed the top 20 GO terms that were enriched in ovary samples collected 1 day after eyestalk ablation, which included transmembrane transporter activity, protein folding, nucleoside monophosphate biosynthetic process, and nucleoside monophosphate metabolic process. Figure 3B showed the top 20 GO terms that were enriched in ovary samples collected 6 days after eyestalk ablation, which included aspartic-type endopeptidase activity, aspartic-type peptidase activity, synapse organization, and cell junction organization. Finally, Figure 3C showed the top 20 GO terms that were enriched in ovary samples collected 11 days after eyestalk ablation, which included pigment binding, protein homodimerization activity, cellular response to organic cyclic compound, and water-soluble vitamin metabolic process.

Further, Figure 3D–F showed that the top 20 KEGG pathways were significantly enriched in ovary samples collected at different timepoints. The steroid hormone biosynthesis pathway was enriched in the *OvI_vs_SOvI* group at 1 day after eyestalk ablation, and in the *OvII_vs_SOvII* group, 6 days after eyestalk ablation, the estrogen signaling pathway, fatty acid degradation, and the MAPK signaling pathway were enriched, which are closely associated with ovarian development and the early recovery of meiosis in oocytes. At 11 days after eyestalk ablation, the hedgehog signaling pathway was activated, which is related to follicular development and proliferation. Other pathways, including fatty acid biosynthesis, fatty acid elongation, amino sugar and nucleotide sugar metabolism, as well as vitamin digestion and absorption showed obvious enrichment, which seem to support oocyte rapid growth.

### 3.4. GO Terms and KEGG Pathway Analysis of Hepatopancreas Samples

**Figure 4:**
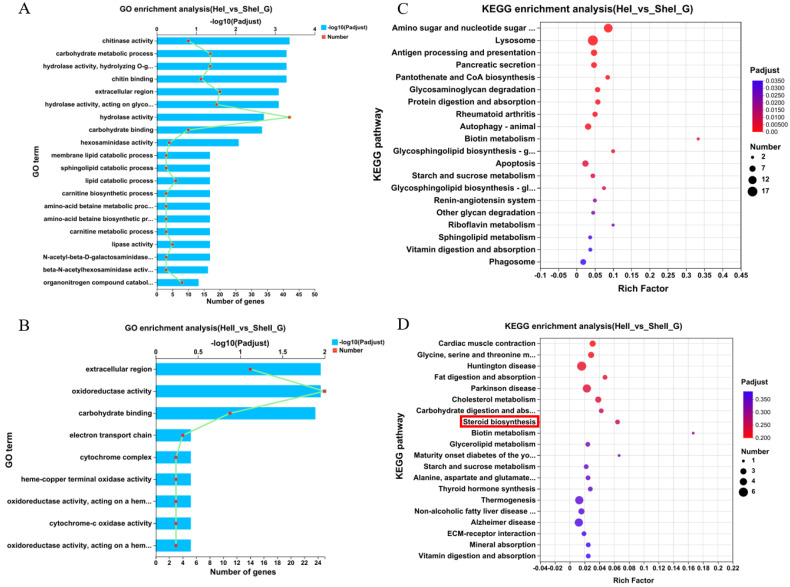
GO and KEGG pathway enrichment analyses of non-redundant transcripts in the hepatopancreas after unilateral eyestalk ablation in *E. carinicauda*. (**A**) Top 20 enriched biological processes in *HeI_vs_SHeI* and (**B**) *HeII_vs_SHeII*. (**C**) Top 20 significant enriched KEGG pathways in *HeI_vs_SHeI* and (**D**) *HeII_vs_SHeII*.

Figure 4A,B showed the top 20 GO terms that were enriched in hepatopancreas samples collected 1 day after eyestalk ablation (e.g., hydrolase activity, carbohydrate activity, and extracellular region) and 6 days after eyestalk ablation (e.g., oxidoreductase activity, extracellular region, and carbohydrate binding), respectively. Further, Figure 4C,D showed that the top 20 KEGG pathways were significantly enriched in hepatopancreas samples collected at different timepoints. Amino sugar and nucleotide sugar metabolism, lysosome, and antigen processing and presentation were enriched in the *HeI_vs_SHeI* group at 1 day after eyestalk ablation, while steroid biogenesis and fat digestion and absorption were enriched in *the HeII_vs_SHeII* group at 6 days after eyestalk ablation.

### 3.5. Identification and Verification of Co-Expressed DEGs

**Figure 5:**
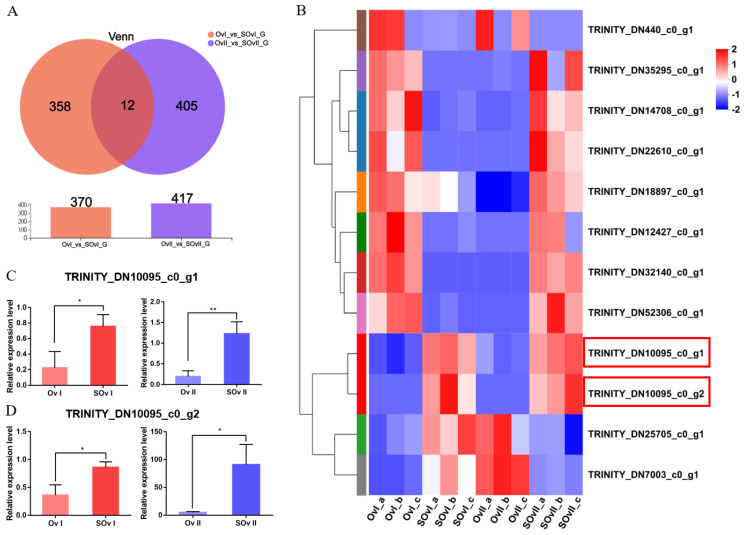
DEGs in the ovary. (**A**) Venn diagram of DEGs at 1 and 6 days after eyestalk ablation in *E. carinicauda*. (**B**) Heatmap of the 12 overlapping genes. (**C**) Validation of TRINITY_DN10095_c0_g1 and (**D**) TRINITY_DN10095_c0_g2 expression levels by real-time PCR at different timepoints (* *p* < 0.05, ** *p* < 0.01).

**Figure 6:**
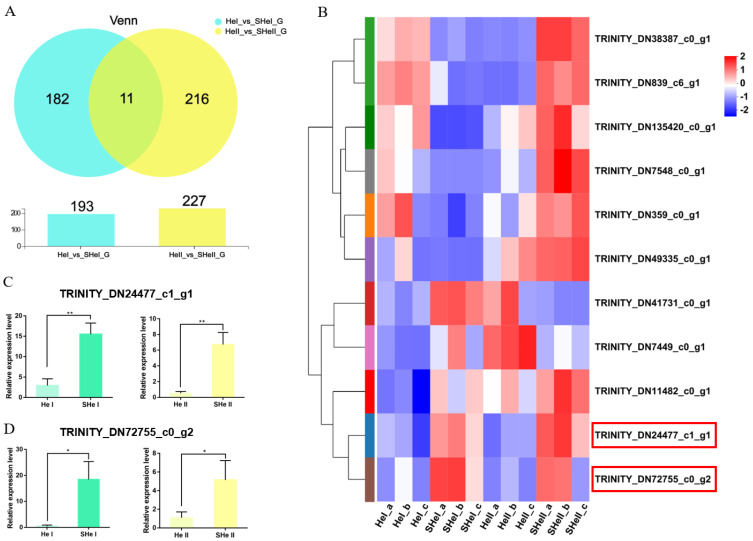
DEGs in the hepatopancreas. (**A**) Venn diagram of DEGs at 1 and 6 days after eyestalk ablation in *E. carinicauda*. (**B**) Heatmap of the 11 overlapping genes. (**C**) Validation of TRINITY_DN24477_c1_g1 and (**D**) TRINITY_DN72755_c1_g1 expression levels by real-time PCR at different timepoints (* *p* < 0.05, ** *p* < 0.01).

To identify genes related to the early development of the ovary, we performed overlap analyses using the *OvI_vs_SOvI* and *OvII_vs_SOvII* gene sets, followed by cluster analyses. Twelve DEGs were found to be potentially related to ovarian development during the early stage (Figure 5A,B). TRINITY_DN10095_c0_g1 and TRINITY_DN10095_c0_g2 were focused as the expression level was higher after eyestalk ablation which might play roles during ovary development. Meanwhile, 11 DEGs were identified on overlap analyses using the *HeI_vs_SheI* and *HeII_vs_SheII* gene sets (Figure 6A,B). The expression of TRIN-281ITY_DN24477_c1_g1 and TRINITY_DN72755_c0_g2 was also upregulated after eyestalk ablation. The expression of the four transcripts was verified by real-time PCR (Figure 5C,D and Figure 6C,D), and were found to be consistent with transcriptome data. However, the specific function of these genes needs to be further determined using other experimental methods.

### 3.6. STEM Analysis of the Expression Profiles of DEGs in Ovary Samples by Time Course

**Figure 7:**
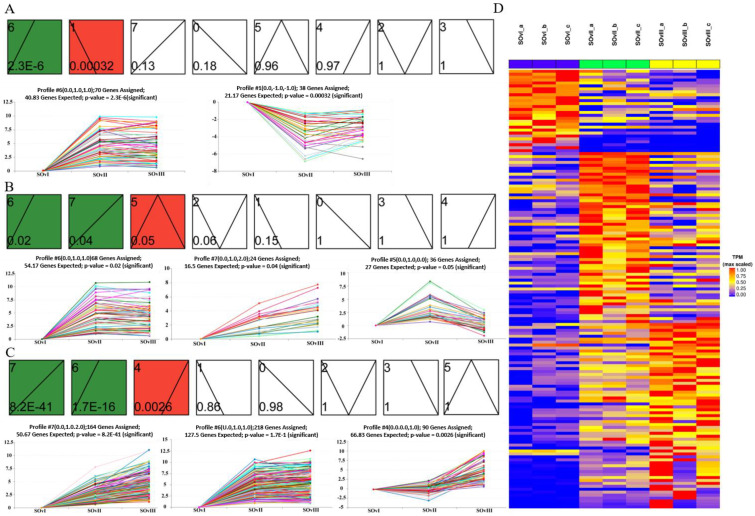
STEM analysis to assess temporal expression trend of DEGs in the ovary at different timepoints. (**A**) Two, (**B**) three, and (**C**) three temporal expression profiles of DEGs from *OvI_vs_SOvI*, *OvII_vs_SOvII*, and *OvIII_vs_SOvIII*, respectively. (**D**) Heatmap of DEGs from different groups of ovaries.

Overall, 149 DEGs were identified by misSigPro based on time course on comparing eyestalk ablation and control groups; Figure 7D showed a heatmap of the cluster of DEGs. These DEGs were then subjected to STEM analysis according to expression levels, which resulted in eight clusters. Each cluster showed a specific pattern, such as gradually increasing or decreasing expression over time. There were two clusters in the *OvI_vs_SOvI* group (Figure 7A), three in the *OvII_vs_SOvII* group (Figure 7B), and three in the *OvIII_vs_SOvIII* group (Figure 7C).

### 3.7. Cluster Analysis and Enrichment of DEGs in Hepatopancreas Samples by Time Course

**Figure 8:**
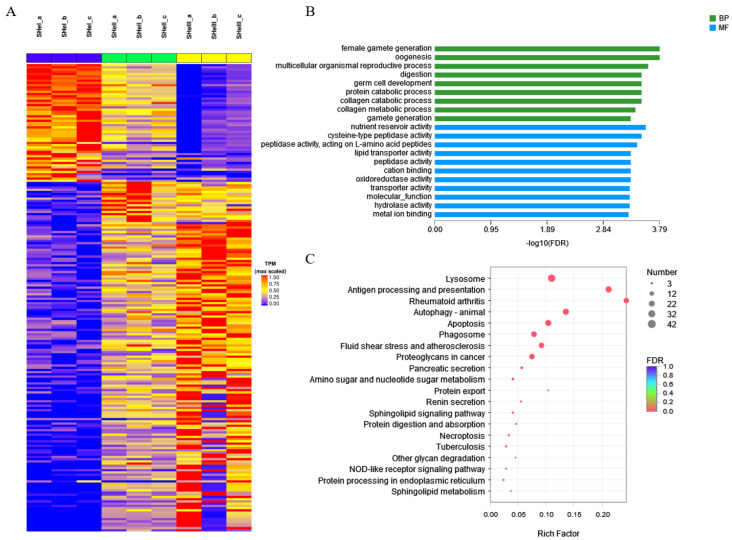
STEM analysis of DEGs in the hepatopancreas. (**A**) Heatmap of DEGs from different groups of the hepatopancreas. (**B**) GO and (**C**) KEGG pathway enrichment analysis of DEGs by time course.

Overall, 210 DEGs were identified by misSigPro based on time course on comparing eyestalk ablation and control groups; Figure 8A showed a heatmap of the cluster of DEGs. Figure 8B showed the results of GO enrichment analysis performed using the top 20 terms. The GO terms related to ovarian development were female gamete generation, oogenesis, multicellular organismal reproductive process, germ cell development, and gamete generation, indicating that the hepatopancreas played a role in ovarian development and maturation. The other GO terms were related to the metabolizing function of the hepatopancreas, such as digestion, protein catabolic process, collagen catabolic process, collagen metabolic process, nutrient reservoir activity, lipid transporter activity, hydrolase activity, and metal ion binding. The top 20 enriched KEGG pathways were shown in Figure 8C. The sphingolipid signaling and sphingolipid metabolism pathways were found to be enriched. These pathways were evidently associated with catalyzing the conversion of sphingomyelin to ceramide. Ceramide acts as a second messenger in regulating oocyte development.

### 3.8. Dmrt1 and cyp307a1 Expression Profile in the Ovary by Two-Color Fluorescent In Situ Hybridization

**Figure 9:**
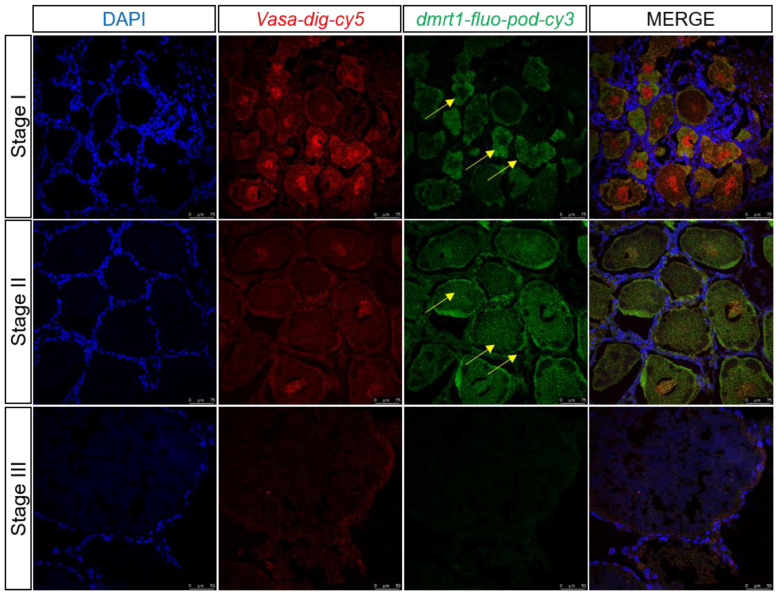
Two-color fluorescent in situ hybridization showing expression profiles of *dmrt1* and *vasa* in the ovary at different stages (scale, Stage I, II: 75 μm; Stage III: 50 μm). Blue signal: DAPI; red signal: *vasa*; and green signal: *dmrt1*.The yellow arrow marks the green signal.

**Figure 10:**
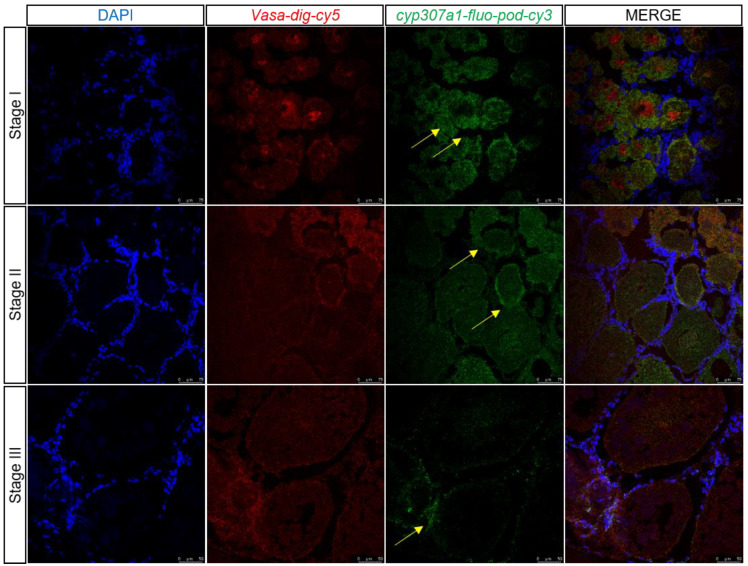
Two-color fluorescent in situ hybridization showing expression profiles of *cyp307a1* and *vasa* in the ovary at different stages (scale, Stage I, II: 75 μm; Stage III: 50 μm). Blue signal: DAPI; red signal: *vasa*; and green signal: *cyp307a1*. The yellow arrow marks the green signal.

During transcriptome data analysis, *dmrt1* and *cyp307a1* were found to be expressed highly in germ cells which were marked by *vasa* at 1 and 6 days after eyestalk ablation and expressed little at 11 days after eyestalk ablation. Two-color fluorescent in situ hybridization was performed to detect *dmrt1* and *cyp307a1* expression profile in samples of ovary at different stages (Figure 9 and Figure 10). We observed that *dmrt1* was mainly expressed during the early development stage (Stage I). This finding was consistent with our transcriptome data. *cyp307a1* was mainly highly expressed during Stage I and Stage II and less so in mature oocyte (Stage III). As a transcription factor, *dmrt1* has the ability to target specific DNA sequences and affect gene expression. We hope to use *dmrt1* as a clue to search for more genes related to early development stage. Based on our findings, we believed that *dmrt1* might play a key role in regulating genes related to ovary development and *cyp307a1* might function during oocyte development.

## 4. Discussion

In this study, we performed transcriptome sequencing to analyze the gene expression profile in *E. carinicauda* ovary and hepatopancreas tissues at different timepoints after eyestalk ablation. To our knowledge, we for the first time reported the DEG expression profile in the *E. carinicauda* hepatopancreas; moreover, STEM analysis was performed to assess the expression trend of DEGs, providing a foundation for the further study of genes with some specific expression trends during ovary development.

In crustaceans, the eyestalk has a key role in diverse physiological activities, such as reproduction, molting, carbohydrate and lipid metabolism, and hydromineral regulation [15,16,17]. Upon eyestalk ablation, the secretion of GIH/MIH is reduced; therefore, its inhibitory effect on ovarian development is weakened [18]. Consequently, the pace of ovarian development and maturation is accelerated. Our results suggested that in response to eyestalk ablation, the gonadosomatic index showed an obvious increase. Further, we observed rapid development of the ovary from oogonia eyestalk ablation (Figure 1). Similar to our findings, eyestalk ablation had been previously used to promote ovarian development in other species. A study evaluated the effects of bilateral and unilateral eyestalk ablation on VTG synthesis in *Marsupenaeus japonicus* to report that after bilateral eyestalk ablation, ovarian development was faster than the ones with unilateral eyestalk ablation. However, in production practice, spawning was promoted usually by unilateral eyestalk ablation since bilateral eyestalk ablation could cause high mortality [19]. Another study found that an inflammatory reaction was induced upon eyestalk ablation, indicative of the activation of the inflammatory response pathway [20]. Even in black tiger shrimp and *Macrobrachium nipponense* [16,17,21], ovarian development was reportedly also promoted by eyestalk ablation.

There were several reports about eyestalk ablation in other crustaceans but the focus was different. Liu et al. investigated the impact of eyestalk ablation on the transcriptomic responses of three major nervous organs including the eyestalk ganglion, brain and thoracic ganglion [22]. Their study mainly concentrated on the nervous system and hormone. Wang et al. focused on the transcripts related to reproduction in eyestalk and ovary during five different timepoints in *Litopenaeus vannamei* [21]. These was a similar case with different methods. Umaporn et al. employed cDNA microarray of ovary to examine effects of eyestalk ablation in black tiger shrimp [20]. Kanchana et al. found that proper nutrient intake was essential for ovary development as well as eyestalk ablation in black tiger shrimp [23]. Contrasted with the previous study, our result focused on the genes and pathways related to ovary early development in the ovary and the hepatopancreas after eyestalk ablation in *E. carinicauda*. Our study first reported the gene profiles in the hepatopancreas after eyestalk ablation in *E. carinicauda*.

The KEGG pathway analysis showed that several key pathways related to ovary development were significantly regulated after eyestalk ablation, including steroid hormone biosynthesis, the estrogen signaling pathway, fatty acid degradation, the MAPK signaling pathway, the hedgehog signaling pathway and steroid biosynthesis. It was reported that the steroid hormone biosynthesis pathway was essential for Chinese sturgeon ovary development and it could be facilitated by adding 14% dietary lipid. [24]. The estrogen signaling pathway had multiple roles during vertebrate fetal development including reproduction [25]. The fatty acids including palmitic, stearic, oleic, and linoleic fatty acids were consistently the most prevalent in oocytes across species and they could provide energy production for ovarian somatic cells and oocytes [26]. The hedgehog signaling pathway played a role in follicle cells and could determine how the other pathway modulated follicle development in mice [27]. In addition, the steroid biosynthesis pathway was also reported to play roles in ovary development [28]. Most steroid hormones could be secreted by gonads and the released into blood to regulate organ development. In our study, these pathways were enriched from 1daa to 11daa. Thus, we could infer that the eyestalk ablation caused the change in hormones level and then the related pathway was regulated. Then, the energy and substance-related to oogenesis were prepared to promote the ovary development. Our findings could offer clues for further study the molecular mechanisms of ovary development.

In non-mammalian vertebrates, follicular cells could secrete androgen, which initiates VTG synthesis in the liver; once secreted, VTG is transported to the ovary by blood and hydrolyzed to yolk proteins [29]. Two models have been reported to explain the process of vitellogenesis in fish: the “single *vtg* model” and “multiple *vtg* model” The latter model describes vitellogenesis more accurately and is thus widely accepted [30]. Yolk proteins in fish are degraded into three core components, Lv, Pv and β′-c [30]. In crustaceans, the gonad and hepatopancreatic index significantly change during vitellogenesis, being closely related to the synthesis of VTG in large quantities. In our previous study, we observed that the concentration of VTG in *E. carinicauda* hemolymph increased at first and then decreased during the entire period of ovarian development [31]. The hepatopancreas has a key role in this process. Notably, in this study, STEM analysis showed eight clusters in hepatopancreas samples after eyestalk ablation (Supplementary material Figure S1), which suggested that several genes were differentially expressed in the hepatopancreas during the vitellogenesis process in crustaceans, in general. Furthermore, GO enrichment analyses revealed two terms related to ovarian development: female gamete generation and oogenesis. Collectively, these findings could provide clues to further explore signaling molecules of vitellogenesis initiation in the hepatopancreas in crustacean.

*dmrt1* plays a vital role in vertebrate sex differentiation and is expressed in both the testis and ovary [32]. The molecular mechanism of *dmrt1* in the testis is well established; *dmrt1* regulates testis differentiation by binding to *sox9*, a male differentiation marker [33]. *dmrt1* mutation in medaka causes male-to-female sex reversal after sex determination [34]. Briefly, *dmrt1* promotes male differentiation by activating the male pathway and antagonizing the female pathway [35,36]. However, only a few studies have investigated the function of *dmrt1* in the ovary. It was reported that *dmrt1* could promote early oogenesis by transcriptional activation of Stra8 in the mammalian fetal ovary [37,38]. Herein, we analyzed *dmrt1* expression levels during different periods of ovarian development in *E. carinicauda* and found *dmrt1* expression to be markedly upregulated in the early stage than in the later stages (II and III). Two-color fluorescent in situ hybridization results validated this finding (Figure 9). Therefore, we believed that *dmrt1* was crucial for oogenesis in *E. carinicauda*. However, further studies are needed to employ such as ChIP-seq and dual-luciferase assay, to determine whether *dmrt1* regulates oogenesis by activating *stra8*.

These findings suggested that the ovary development could be promoted by eyestalk ablation in *E. carinicauda*. The hepatopancreas was an important organ during vitellogenesis and *dmrt1* might play roles in ovary early development by arising meiosis.

## 5. Conclusions

To summarize, we reported comparative changes in *E. carinicauda* ovary and hepatopancreas after eyestalk ablation at the transcriptome level for the first time. Based on our transcriptome data and verification results, the transcripts TRINITY_DN10095_c0_g1, TRINITY_DN10095_c0_g2, and *dmrt1* in the ovary as well as TRINITY_DN24477_c1_g1 and TRINITY_DN72755_c0_g2 in the hepatopancreas seemed to play a crucial role in ovary development, including vitellogenesis and oogenesis. Our findings should support future studies focusing on exploring oogenesis and ovarian development in crustaceans.

## Data Availability

The datasets presented in this study can be found in online repositories. The data presented in this study are deposited in the NCBI SRA repository with accession number PRJNA863229.

## Supplementary Materials

The following supporting information can be downloaded at: https://www.mdpi.com/article/10.3390/biology12040596/s1, Table S1: The notation of the 11 transcripts in hepatopancreas; Table S2:The annotation results for each transcript by six databases; Table S3: Quality Evaluation of the De novo transcriptome data; Table S4: Primer sequence; Table S5: The annotation results for 6 Databases; Figure S1: The clusters in hepatopancreas by time course.
