# Supplementary material for: Molecular Characterization Related to Ovary Early Development Mechanisms after Eyestalk Ablation in Exopalaemon carinicauda
Source: Biology (Basel). 2023 Apr 14;12(4):596. doi: 10.3390/biology12040596 (PMC10135610; doi:10.3390/biology12040596)

Table S3. Quality Evaluation of the De novo transcriptome data

Sample	Clean reads	Clean bases	Error rate(%)	Q20(%)	Q30(%)	GC content(%)
OvI_a	55882126	8229364050	0.0246	98.22	94.50	42.29
OvI_b	55613822	8201873937	0.0248	98.14	94.28	41.97
OvI_c	48445080	7146729229	0.0247	98.19	94.37	42.31
SOvI_a	52896924	7820313970	0.0247	98.19	94.38	42.57
SOvI_b	53701738	7931764637	0.0245	98.25	94.56	42.90
SOvI_c	45767490	6745065273	0.0246	98.21	94.45	42.78
HeI_a	48711120	7221628208	0.0248	98.16	94.30	42.68
HeI_b	43227492	6433246045	0.0248	98.12	94.27	43.17
HeI_c	45423164	6766675750	0.0247	98.18	94.42	42.94
SHeI_c	51777750	7678478304	0.0242	98.40	94.93	45.57
SHeI_b	52047526	7703427907	0.0244	98.32	94.72	43.77
SHeI_a	46778930	6929753120	0.0246	98.24	94.55	44.82
OvII_a	53622864	7913738036	0.0248	98.16	94.31	42.19
OvII_b	52055498	7692270434	0.0245	98.27	94.61	42.78
OvII_c	48824246	7235695862	0.0245	98.25	94.59	42.65
SOvII_a	53605160	7861224617	0.0247	98.18	94.37	42.11
SOvII_b	47153710	6975717993	0.0245	98.28	94.64	43.73
SOvII_c	57598794	8486354462	0.0246	98.23	94.52	42.07
HeII_a	49056286	7290180246	0.0245	98.25	94.60	44.96
HeII_b	50490098	7467213428	0.0242	98.40	94.95	44.80
HeII_c	48228518	7145561673	0.0243	98.34	94.81	43.45
SHeII_a	52385238	7759887937	0.0242	98.37	94.86	44.32
SHeII_b	56163690	8294010626	0.0243	98.34	94.80	44.45
SHeII_c	53059352	7886558574	0.0242	98.39	94.89	44.79
OvIII_a	53646032	7944327277	0.0249	98.12	94.23	43.26
OvIII_b	50121396	7371963365	0.0244	98.29	94.68	42.29
OvIII_c	47597456	7074079301	0.0246	98.23	94.52	42.08
SOvIII_a	42288610	6257732949	0.0249	98.11	94.21	41.47
SOvIII_b	50878952	7528949373	0.0246	98.21	94.46	41.49
SOvIII_c	44346934	6587805554	0.0270	97.33	92.12	43.04
SHeIII_a	46044598	6844789384	0.0246	98.24	94.52	43.72
SHeIII_b	45249944	6720891232	0.0246	98.20	94.47	44.06
SHeIII_c	50526254	7500713045	0.0243	98.36	94.80	44.00

Table S4. Primer sequence

primer name	Gene ID	primer sequence(5'to3')	primer usage
1-F	TRINITY_DN10095_c0_g1	CTTCCTGACGAGGCGGTGAC	*real-time* PCR
1-R		TGCCTTGGCAAGAAGCCAAA	
2-F	TRINITY_DN10095_c0_g2	GAGGGCTTCAGCTTCCTGCC	
2-R		CGAAGTCACCGCCTCGTCAG	
3-F	TRINITY_DN24477_c1_g1	GCCTTTGCCACCTTATACCA	
3-R		TGGTAAGAGACTCTGCGTCA	
4-F	TRINITY_DN72755_c0_g2	GTCTTGCCCAGTGGATTTCA	
4-R		GCTTCATGTCGTACCAGGAG	
18S-F	18S	TGTATCTCAGGGCCTTGTCT	
18S-F		GACGTTTCCTGTACCTGGAC	
Dmrt1-F	*Dmrt1*	CCGTGAAAACTCGAGAGGTCCGC	*in-situ hybridization*
Dmrt1-R		TAATACGACTCACTATAGGC GTGAGGCACCGAGGCAAGG	
Vasa-F	*Vasa*	GCCGTTGCTCACAAGGTGGC	
Vasa-R		TAATACGACTCACTATAGG GGGGACGGGAGAGGGAGAGG	
Cyp307a1-F	*Cyp307a1*	GGAGCGCTTGCCTGTGCTGA	
Cyp307a1-R		TAATACGACTCACTATAGGTTCCGCG GCACCGAATCCAC	

Table S5.The annotation results for 6 Database

Database	Exp_Unigene number(%)	Exp_Transcript number(%)	All_Unigene number(%)	All_Transcript number(%)
GO	17004(0.1747)	30942(0.1624)	17006(0.1746)	30951(0.1623)
KEGG	12237(0.1257)	22097(0.1159)	12237(0.1257)	22105(0.1159)
eggNOG	16652(0.1711)	29866(0.1567)	16652(0.171)	29876(0.1566)
NR	22286(0.229)	40654(0.2133)	22288(0.2289)	40669(0.2132)
Swiss-Prot	14588(0.1499)	25992(0.1364)	14588(0.1498)	26001(0.1363)
Pfam	17578(0.1806)	31416(0.1648)	17580(0.1805)	31427(0.1647)
Total_anno	24032(0.2469)	43698(0.2293)	24034(0.2468)	43714(0.2292)
Total	97319(1)	190575(1)	97383(1)	190757(1)

Figure S1 The clusters in hepatopancreas by time course

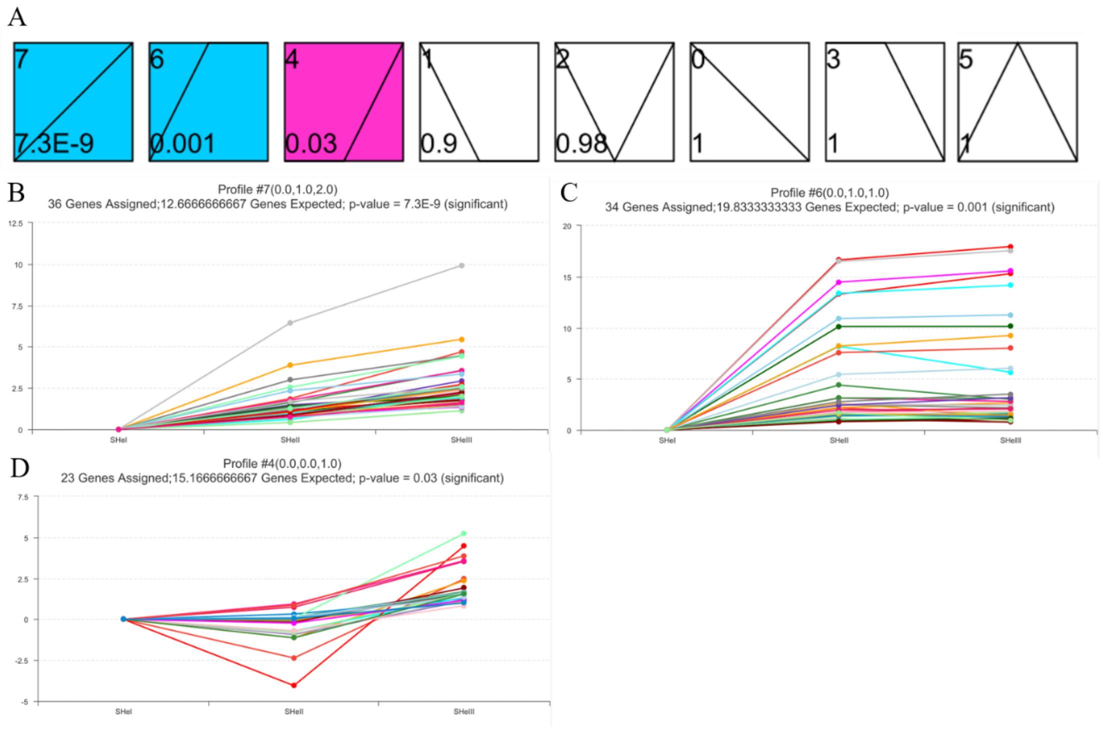

Supplement: Supplementary file 1 [file biology-12-00596-s001.zip › revision----Supplementary material.pdf]
